# Do sociodemographic factors influence the levels of health and oral literacy? A cross-sectional study

**DOI:** 10.1186/s12889-023-17489-4

**Published:** 2023-12-20

**Authors:** Francisco Manuel Veigas Veladas, Giancarlo De la Torre Canales, Bryanne Brissian de Souza Nobre, Ana Escoval, Ana Rita Pedro, André Mariz de Almeida, Victor Abreu Assunção, Ana Cristina Manso

**Affiliations:** 1 Egas Moniz Center for Interdisciplinary Research (CiiEM), Egas Moniz School of Health & Science, Almada, Caparica Portugal; 2 Department of Dentistry, Ingá University Center, Uningá, Maringá, Paraná Brazil; 3 Division of Oral Diagnostics and Rehabilitation, Department of Dental Medicine, Karolinska Institutet, Huddinge, SE-14104 Sweden; 4 Escola Nacional de Saúde Pública NOVA, Centro de Investigação Em Saúde Pública, Centro de Investigação Em Saúde Compreensiva, CHRC, Universidade NOVA de Lisboa, Lisbon, Portugal; 5 Faculdade de Medicina Dentária, Universidade de Lisboa, Lisbon, Portugal

**Keywords:** Oral Health Literacy, Health Literacy, Sociodemographic factors

## Abstract

**Background:**

Oral health literacy has gained importance in dental literature, and its relationship with oral health status and association with health status (HL) has been reported. Then, an association between the levels of HL and OHL could be expected. This study aimed to assess the levels of HL and OHL according to sociodemographic factors and to explore a possible association between HL and OHL.

**Methods:**

The European Health Literacy Survey and Oral Health Literacy Adults Questionnaire were applied to a convenience sample from Portuguese individuals. Also, sociodemographic factors such as sex, age, schooling level of the participants and their parents, and if the participants were professionals or students of the health field were assessed. To analyze the data, the Kruskal–Wallis and Mann–Whitney U tests were used to compared sociodemographic variables and the levels of literacy in general and oral health. The Spearman correlation test assessed the correlation between the levels of HL and OHL.

**Results:**

HL results showed that 45.1% of the volunteers were considered in a “problematic level” and 10.3% in “excellent level”. However, 75% presented an adequate level of OHL. Regarding the levels of HL in each sociodemographic variable, significant higher levels of “excellent level” were found in health professionals and students when compared with participants not related to health area (*p* < 0.001). Comparisons between the levels of OHL in each sociodemographic variable showed, significant differences regarding sex (*p* < 0.05), age (*p* < 0.001), levels of schooling of the participants and their parents (*p* < 0.009 and *p* < 0.001) and relationship with health field. (*p* < 0.001). A significant positive – weak correlation was found between HL and OHL (*p* < 0.001).

**Conclusions:**

HL and OHL levels are associated and could be influenced by sociodemographic factors.

## Background

Numerous definitions of health literacy (HL) have been proposed [1, 2] Notwithstanding, almost all definitions embrace the same elements, which describe a set of observable literacy skills that allow individuals to obtain, understand, appraise, and use information to make decisions and take actions that will influence health status, which vary from individual to individual [3, 4]. Therefore, limited HL represents an important challenge for health policies and practices across the world, since poor levels of health literacy makes difficult to read, understand and apply for health information (e.g., wording on medication bottles, discharge instructions, informed consent documents, insurance applications, and health education materials) [3].

In the United States, the 2003 National Assessment of Adult Literacy (NAAL) reported that 36% of the U.S. adult population has a basic or below basic HL [3]. On the other hand, the European Health Literacy Project (HLS-EU) which consisted of nine organizations from eight European Union (EU) member states, reported a 12.4% of inadequate HL, with substantial differences between members states [2]. Taking together, these results pointed out the existence of specific vulnerable groups which are influenced by sociodemographic variables [4]. In addition, the HSL-EU showed that financial deprivation, social status, education, age, and genders are predictors of limited HL [2].

In this scenario, Oral health literacy [5] has gained importance in dental literature in the last decade [6]. Studies have concluded that OHL is crucial in diminishing oral health disparities and in promoting oral health [7]. On the other hand, populations with limited OHL have elevated risk to develop oral diseases [6], problems with the use of preventive services, poor adherence to medical instructions and self-management skills, higher health care costs and higher mortality risks [8, 9]. Furthermore, it was demonstrated by the Carolina Oral Health Literacy (COHL) study together with other reports, the strong influence of OHL in health behaviors and outcomes [10–12]. Notwithstanding, a systematic review assessing the scientific evidence regarding the association between OHL and oral conditions, concluded that the evidence is weak, and that this association remains unsubstantiated, mainly because of the low quality of the available studies. However, it has been pointed out that health-related decisions made by people influence their health, which is also influenced by health literacy, and modulated by sociodemographic factors. Also, it have been explained that a relationship between OHL and health status exists [13], and that OHL is associated with oral health status [6]. Therefore, it could be hypothesized that there is also an association between the levels of HL and OHL, since health determinants like income, education and personal characteristics influence health behaviors and oral health outcomes [6].

Thus, the aim of this study was to assess the levels of HL and OHL according to sociodemographic factors and to explore a possible association between the degrees of HL and OHL.

## Methods

This research was approved by the Research Ethics Committee of Egas Moniz School of Health and Science, Almada, Portugal (N°: 1078) and conducted in accordance with the ethical principles of the Declaration of Helsinki. All individuals were informed about the research purposes and signed a voluntary informed consent form. This observational cross-sectional study was conducted following the recommendations of the Strengthening the Reporting of Observational Studies in Epidemiology (Strobe) guidelines [14].

The convenience sample was obtained from Portuguese individuals, over 16 years old. Data collection was conducted from May 24 to June 21, 2022, by using an online form, via Google Forms (Google; Mountain View, CA, USA). Briefly, the first page of the online questionnaire presented the Informed Consent Form, which described the research aims and potential risks and benefits. Thus, volunteers who accepted to participate in the study were required to digitally sign the Inform Consent Form before proceeding to fill out the structured questionnaires. The average time to fill out the entire questionnaire was approximately 12 min. Participants were invited to participate in the study by email and Whatsapp®, from which they received a link to access to the complete online form.

### Health, oral health levels and sociodemographic factors

#### European Health Literacy Survey (HLS-EU-PT-Q16 short version)

The HLS-EU-PT-Q16 consists of 16 questions based on 3 domains, embracing health care, health promotion and disease prevention. Using a 4-point scale, the survey rates the degree of difficulty in carrying out tasks related to each domain. Then, a score is obtained by summing up the answers (0 to 50), in order to metric standardized the level of health literacy in four levels, depending on the score obtained: inadequate (0 to 25), problematic (25 and 33), sufficient (33 and 42) and excellent (42 to 50) [15, 16]

#### Oral Health Literacy Adults Questionnaire (OHL-AQ)

The OHL-AQ is composed of 17 items divided into 4 different sections: reading comprehension, numeracy, active listening, and decision-making. The total score (0 to 17) is obtained through the sum of all the questions answered correctly, which are given a score of one. Thus, the total score is categorized into three different levels: inadequate (0 to 9), marginal (10 and 11) and adequate (12 to 17) [17, 18]

#### Sociodemographic factors

To obtain a detailed characterization of the studied sample the following sociodemographic characteristics were assessed: sex, age, schooling level of the participants and their parents, and if the participants were health professionals or students of the health field.

### Statistical analysis

The data collected on the digital platform was exported and tabulated. Descriptive statistics were performed to identify frequencies and distributions of the outcomes. Since the data presented no normal distribution, the Kruskal–Wallis and Mann–Whitney U tests were used to compare sociodemographic variables and the levels of literacy in general and oral health. The correlation between the levels of literacy in health and in oral health was assessed by the Spearman correlation test. Analyses were performed with SPSS software, version 28.0 (IBM Statistical Package for Social Sciences) with a 5% significance level.

## Results

A total of 205 participant’s answers were obtained in our study. However, since the HSL-EU-EN-Q16 questionnaire is considered valid when at least 80% of its questions have been answered, our study considered a total of 204 of valid questionnaires. The mean age of the studied population was 30.6 (± 6.3). Most of the participants included were females and the group related with health area was composed mainly of students (70%). Participant’s distribution according to sociodemographic factors are shown in Table 1.

**Table 1:** Demographic characteristics of the studied sample

	(n)	%
*Sex*		
Female	164	80,4
Male	40	19,6
*Age*		
16—24	107	52,5
25—39	32	15,7
≥ 40	65	31,9
*Schooling Level*		
Basic / High school	48	23,5
Superior	156	76,5
*Profissional / Student in Health Area*		
Yes	107	52,5
No	97	47,5
*Parent’s schooling*		
Basic	57	27,9
High School	56	27,5
Superior	91	44,6

**Fig. 1:**
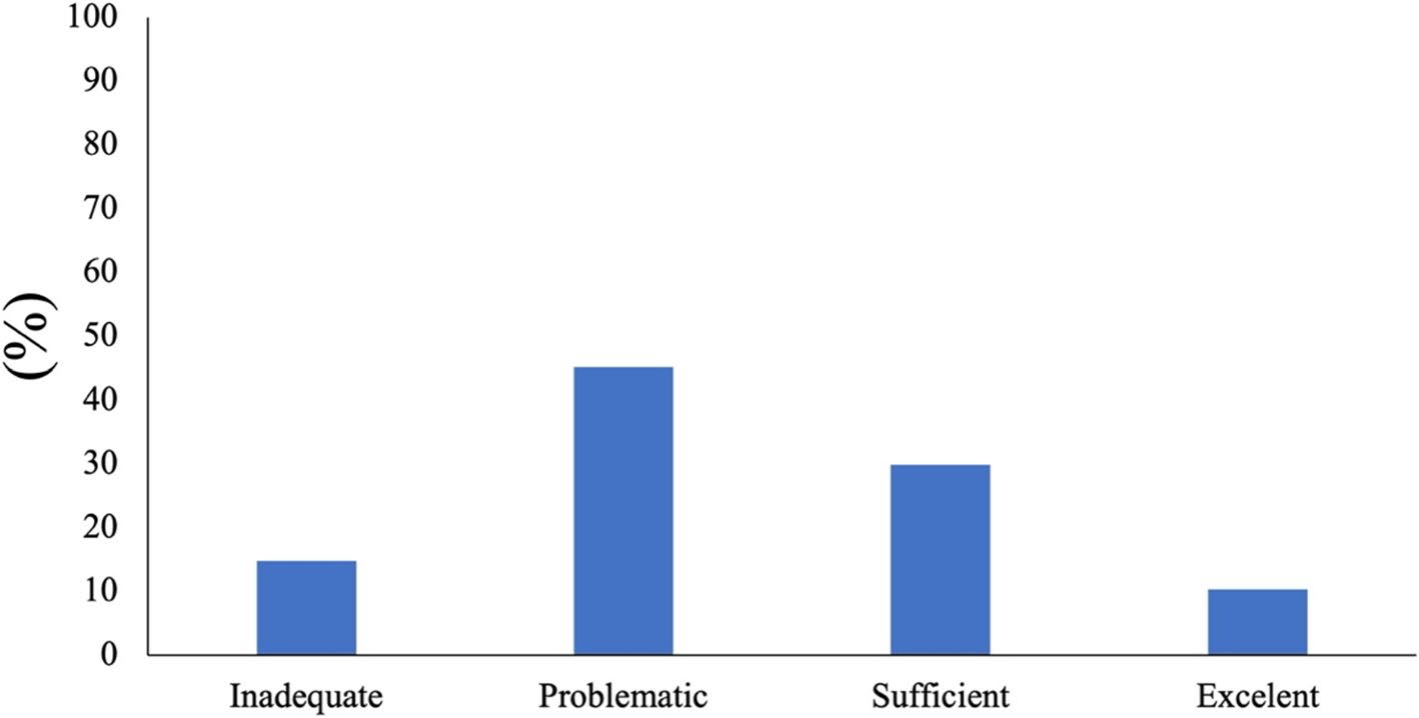
General Health Literacy frequencies in the studied population

**Fig. 2:**
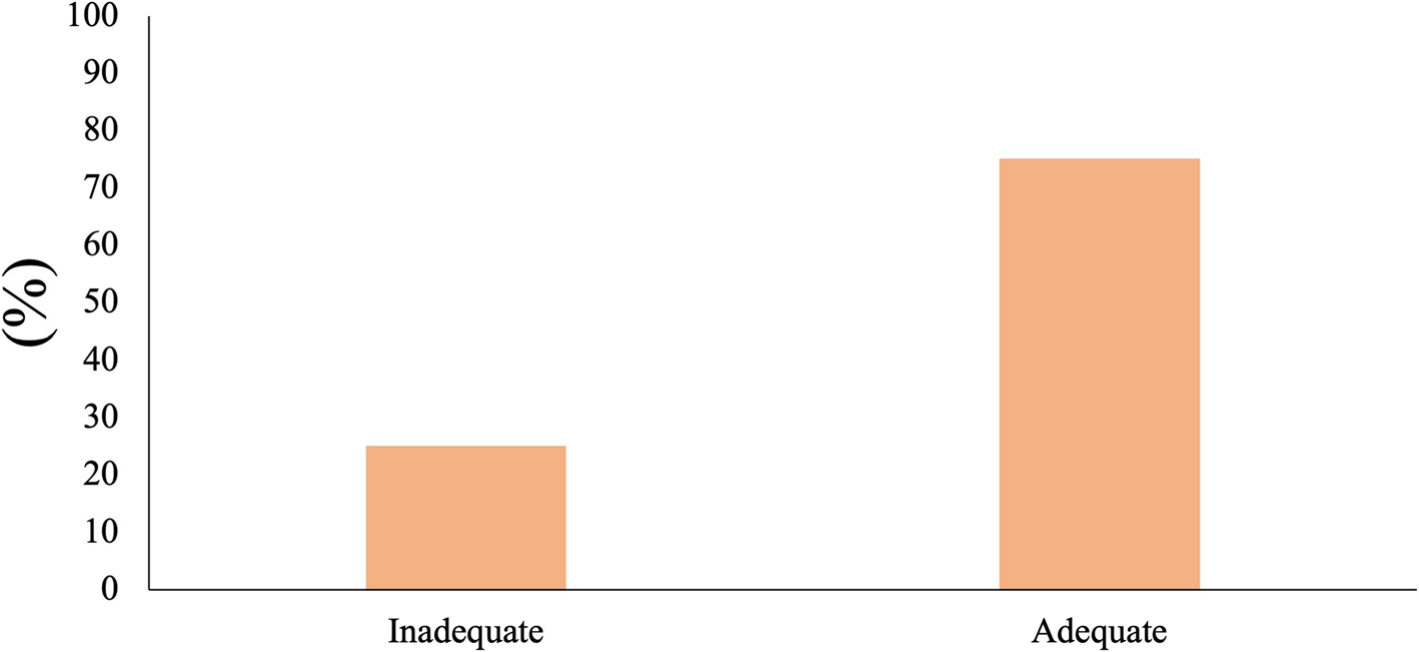
Oral Health Literacy frequencies in the studied population

Regarding the levels of health literacy 45.1% of the volunteers were considered in a “problematic level”, 29.9% in “sufficient level, 14.7% in “inadequate level”, and 10.3% in “excellent level” (Fig. 1). On the other hand, most of the participants (75%) presented an adequate level of oral health literacy, while 25% presented an inadequate level (Fig. 2).

The comparisons between the levels of health literacy in each sociodemographic variable (Table 2) showed no significant differences regarding sex (*p* > 0.91), age (*p* > 0.94), schooling level (*p* > 0.24) and parent’s schooling level (*p* > 0.19). However, professionals or students in health area showed higher frequencies of “excellent level” in health literacy when compared with participants that were not related to health area (*p* < 0.001). In addition, participants not related to the health area showed greater frequencies of “inadequate level” when compared with professional or students in health area (*p* < 0.001).

**Table 2:** Distribution (%) and comparisons of General Health Literacy levels considering different sociodemographic variables

	%				*p*
	Inadequate	Problematic	Sufficient	Excelent	
*Sex*					
Female	15.2	43.9	31.1	9.8	0.91
Male	12.5	50.0	25.0	12.5	
*Age*					
16—24	16.8	43.0	28.0	12.1	0.94
25—39	6.3	59.4	28.1	6.3	
> 40	15.4	41.5	33.8	9.2	
*Schooling Level*					
Basic or High school	18.8	45.8	27.1	8.3	0.28
Superior	13.5	44.9	30.8	10.9	
*Professional / Student in Health Area*					
Yes	7.5	44.9	32.7	15.0	0.001^*^
No	22.7	45.4	26.8	5.2	
*Parent’s Schooling Level*					
Basic	21.1	45.6	24.6	8.8	0.19
High school	14.3	44.6	32.1	8.9	
Superior	11.0	45.1	31.9	12.1	

^*^p < 0.05

On the other hand, the comparisons between the levels of oral health literacy in each sociodemographic variable (Table 3) showed, significant differences regarding sex, showing that females presented higher frequencies of “adequate level” and lower frequencies of “inadequate level” (*p* < 0.05). Considering that participants were divided in age sub-groups, higher frequencies of “adequate level” were found in all subgroups, and younger participants presented higher values of “adequate level” when compared with the others sub-groups (*p* < 0.001). In the same way, uppermost levels of schooling of the participants and their parents presented higher levels of adequate knowledge of oral health (*p* < 0.009 and *p* < 0.001). Moreover, professional or students in health area showed increased values of “adequate level” when compared with participants that are not related to health area (*p* < 0.001).

**Table 3:** Distribution and comparisons of Oral Health Literacy levels considering different sociodemographic variables

	%		*p*
	Inadequate	Adequate	
*Sex*			
Female	22.6	77.4	0.05
Male	35.0	65.0	
*Age*			
16—24	15.0	85.0	0.001^*^
25—39	25.0	75.0	
> 40	41.5	58.5	
*Schooling Level*			
Basic or High school	41.7	58.3	0.009^*^
Superior	19.9	80.1	
*Professional / Student in Health Area*			
Yes	9.3	90.7	0.001^*^
No	42.3	57.7	
*Parent’s Schooling Level*			
Basic	50.9	49.1	0.001^*^
High school	21.4	78.6	
Superior	11.0	89.0	

^*^ p < 0.05

Considering the correlation between the levels of literacy in health and in oral health (Table 4), a significant positive but weak correlation was found between these two variables (*p* < 0.001).

**Table 4:** Correlation between General Health and Oral Health Literacy scores

		Score Literacia em Saúde	Score Literacia em Saúde Oral
General Health Literacy Score	Rho (Spearman)	1.000	0.22
	*p*		0.001^*^
	*n*	204	204
Oral Health Literacy Score	Rho (Spearman)	0.22	1.00
	*p*	0.001^*^	
	*n*	204	204

^*^ p < 0.05

## Discussion

In spite of the growing attention being paid to health and oral health literacy among European health policymakers, data regarding the status of this variables in Europe remains scarce. Our study found that 14.7% and 25% of the total surveyed population had an inadequate level of HL and OHL, respectively. Also, our study demonstrated that participants relationship with health area increases the frequencies of “excellent level” in HL; and factors like sex, age, schooling levels and relationship with health area rise “adequate level” frequencies in OHL. Furthermore, a significant positive correlation was found between the levels of HL and OHL. 

It is noteworthy that our results regarding inadequate and problematic levels of HL (59.8%) are in line with a previous study in the same population (Portuguese participants) that reported 61% of the assessed sample presented low levels of HL [16] and differ from a study reporting only 30% of low levels of HL [19]. The later study included participants from other countries (Brazil and Angola) which could explain the discordance with our study [19]. The HLS-EU reported frequencies of 12,4% of “inadequate level" of HL as the mean of the total studied sample, which is in line with our study that reported 14.7%. However, considering the frequencies reported by each of the assessed countries in the mentioned study, our results presented higher frequencies of “inadequate level” of HL, when compared with Ireland, Netherlands, Poland, Spain and Germany and lower frequencies when compared with Austria and Bulgaria [2]. Therefore, the considerable proportions of people with inadequate health literacy implies that health literacy deficit is a challenge for public health in European countries. Differences in health programs, health policies and economic conditions could explain these differences.

Regarding the levels of OHL, 75% of our sample presented an “adequate level”, which is in contrast with Almeida et al. (2022) [20], that reported lower frequencies of “adequate levels” of OHL. Certainly, the fact that the mentioned study assessed a population of a different country that ours, with lower levels of schooling and income affected the results. On the other hand, our results are in line with Mendes (2019) [18] and Flynn et al., (2016) [5] which reported higher frequencies of individuals with an “adequate level” of OHL as well. It is of main importance to know the levels of OHL in a population, since studies have concluded that low levels of OHL are associated with poor oral health knowledge, which may influence self-care behavior, capacity of understand health instructions or the importance of preventive dental procedures [21–25]. Besides, higher prevalence of dental caries, periodontal disease and extracted teeth have been reported in individuals with low OHL [26, 27]. However, most of these results come from studies with methodological drawbacks which could question the validity of the results [28].

The HLS-EU have reported that sociodemographic factors like, social status, education, age, and sex could influence low levels of HL. In addition, the authors concluded that financial depravation is the strongest predictor for low HL [2]. Even though our study found that the relationship of the participants with health area influenced the levels of HL, meaning that health professionals or students present higher levels of HL as expected; our study showed that sex, age, education level of the participants and their parents, and the relationship with health area could influence the levels of OHL. In addition, our study found that women presented higher levels of OHL. This was an expected finding since literature have concluded that women seek for dental treatment more than men [29]. Regarding the age of our sample, the youngest participants presented higher frequencies of OHL; it was also an expected result, since nowadays there are more programs for the promotion of oral health in Portugal with special focus on 16–24 age group, which may have led to greater awareness and education in the context of oral health. Considering the results of educational levels and the relationship with health area, greater levels of education and working or studying in health area, allow to acquire more knowledge about oral health and permit to understand in a better way preventive oral health instructions and procedures, which may explain the higher levels of OHL.

As a final remark, as far as we know, our study is the first one in demonstrate a positive correlation despite being weak between both levels of literacy (HL and OHL), meaning that the levels of each one could significantly affect the levels of the other. In this direction, Macek et al., (2010) [13] provide the rationale for including a measure of conceptual health knowledge in future investigations of OHL, presented a new conceptualization of the pathway between HL and oral health and the importance of assessing HL in dental care.

Although this research has obtained important answers on HL and OHL, some limitations should be considered. First, it is important to note that our study used subjective tools to assess HL and OHL and no objective items are included in this tools to measure functional HL and OHL. Second, the data were collected from a small no probabilistic convenience sample, in which most of the participants included in the group related to health area were students. Therefore, our results should be analyzed with caution and not be extrapolated to other samples since the above-mentioned factors certainly influenced the results of the present study. Third, the cross-sectional design of this study prevents it from elaborating on the cause and effect. Finally, the authors strongly recommend that future studies assess whether HL is associated with more detailed measures of oral health care utilization, in studies with larger sample size and no convenience populations.

## Conclusions

It can be concluded that:

- Low levels of HL and high levels of OHL are prevalent in the studied population.
- Sociodemographic factors could influence the levels of HL and OHL.
- HL and OHL levels are associated.

## Data Availability

The datasets used and/or analyzed during the current study are available from the corresponding author on reasonable request.
